# De Witt County Medical Society

**Published:** 1856-03

**Authors:** C. Goodbrake, Z. H. Madden

**Affiliations:** Pres.; Sec.


*De Witt County Medical Society.*

At a meeting of the physicians of De Witt County, held pursuant to previous notice) in the office of Drs. Goodbrake & Edmiston, at Clinton, March 8th, 1856, on motion of Dr. Goodbrake, Dr. Crist, was called to the Chair; and on motion of Dr. Madden, Dr. Goodbrake was appointed Secretary.

On motion of Dr. Goodbrake it was

*Resolved*, That we deem it expedient to proceed to the organization of a County Medical Society.

On motion of Dr. Goodbrake, the Chairman appointed a committee, consisting of Drs. Goodbrake, Stewart, and Wright to prepare a Constitution and By-Laws, with the request to report to this meeting at its afternoon session.

The meeting then adjourned until 2 o'clock, P. M.

AFTERNOON SESSION, 2 o'clock, P. M.

The meeting met as per adjournment, and was called to order by the Chairman, when Dr. Goodbrake, from the committee appointed at the morning session, reported a Constitution and By-Laws, for the government of the Society, which was unanimously adopted.

On motion of Dr. Stewart, the meeting then proceeded to elect officers (in accordance with the Constitution just adopted) for the ensuing year, which resulted in the election of the following gentlemen, viz.:—

Dr. C. Goodbrake, of Clinton, *President.*
" E. Stewart, of Waynesville, *Vice-President.*
" Z. H. Madden, of Clinton, *Secretary.*
" W. W. Adams, of Clinton, *Treasurer.*

Dr. J. B. Hunt, of Waynesville, Dr. J. H. Tyler, of Marion, and John Wright, of Wappello, *Censors.*

The meeting then, by vote, resolved itself into the De Witt County Medical Society.

The President elect, on taking the chair, delivered a short but pertinent address, making some appropriate remarks on the benefits which would accrue, both to physicirns and their patrons, from proper medical organizations.

Dr. Stewart then presented a table of charges or fees, which had been previously prepared, and which was, after an animated discussion, in which most of the members present partook, unamimously adopted.

On motion of Dr. Stewart, it was

*Resolved*, That we adopt the National code of Ethics, and that the members of this Society be governed by the same in all cases.

On motion of Dr. Madden, the Society then proceeded to the election of delegates, which resulted as follows:—

Delegates to the Illinois State Medical Society, Dr. D. O. Crist, Dr. T. K. Edmiston, and Dr. Z. H. Madden.

Delegate to the American Medical Association, Dr. J. H. Tyler.

The President then appointed Drs. Jno. B. Hunt and John Wright, to write each an essay on some medical subject, of their own choice, to be read before the Society at its next meeting.

On motion, the Secretary was requested to furnish a copy of the proceedings of this meeting to the editors of the De Witt Courier and the North-Western Medical and Surgical Journal, with the request to publish the same.

On motion of Dr. Lewis, the Society then adjourned to meet again in quarterly session at Waynesville, on the first Monday in July, at 10 o'clock, A. M.

C. GOODBRAKE, M.D., *Pres.*

Z. H. MADDEN, M.D., *Sec.*

